# B-vitamins, related vitamers, and metabolites in patients with quiescent inflammatory bowel disease and chronic fatigue treated with high dose oral thiamine

**DOI:** 10.1186/s10020-023-00741-3

**Published:** 2023-10-25

**Authors:** Palle Bager, Christian Lodberg Hvas, Mette Mejlby Hansen, Per Ueland, Jens Frederik Dahlerup

**Affiliations:** 1 Department of Hepatology and Gastroenterology, Aarhus University Hospital, Palle Juul-Jensens Boulevard 99, 8200 Aarhus N, Denmark; 2 Bevital AS, Jonas Lies Veg 87, 5021 Bergen, Norway

**Keywords:** Vitamin B, Fatigue, Inflammatory bowel disease, Thiamine

## Abstract

**Background:**

High doses of oral thiamine improve clinical fatigue scores in patients with quiescent inflammatory bowel disease (IBD) and chronic fatigue. In this study we analysed plasma samples obtained in a randomised clinical trial and aimed compare levels of vitamins B1, B2, B3 and B6, and their related vitamers and metabolites in patients with IBD, with or without chronic fatigue and with or without effect of high dose oral thiamine for chronic fatigue.

**Methods:**

Blood samples from patients with fatigue were drawn prior and after thiamine exposure and only once for patients without fatigue. A wide panel of analysis were done at Bevital AS Lab.

**Results:**

Concentration of flavin mononucleotide (FMN) was lower in patients with chronic fatigue compared to patients without fatigue (p = 0.02). Patients with chronic fatigue who reported a positive effect on fatigue after 4 weeks of high dose thiamine treatment had a statistically significantly lower level of riboflavin after thiamine treatment (p = 0.01).

**Conclusion:**

FMN and Riboflavin were associated with chronic fatigue in patients with quiescent IBD. Levels of other B vitamins and metabolites were not significantly different between the investigated groups or related to effect of the thiamine intervention.

*Clinical trial registration*: ClinicalTrials.gov study identifier NCT036347359. Registered 15 August 2018, https://clinicaltrials.gov/study/NCT03634735?cond=Inflammatory%20Bowel%20Diseases&intr=Thiamine&rank=1

## Introduction

High doses of oral thiamine improve clinical scores of fatigue in patients with quiescent inflammatory bowel disease (IBD) and chronic fatigue (Bager et al. 2021). IBD consists of Crohn’s disease and ulcerative colitis. IBD is characterised by chronic inflammation of the gastrointestinal tract with periodic inactive (quiescent disease) and periodic active inflammation (Torres et al. 2017; Le Berre et al. 2023). Chronic fatigue is regarded as elevated fatigue levels with duration of more than 6 months. Fatigue is a frequent and debilitating extraintestinal manifestation of IBD of both active and quiescent disease, but the underlying mechanisms are not fully explored (Kvivik et al. 2023; McGing et al. 2021). Fatigue in both active and quiescent IBD disease has been related to anaemia, iron deficiency, other deficiencies, and current inflammation (Bager et al. 2012; McGing et al. 2021).

Thiamine is essential for carbohydrates metabolism and mitochondrial adenosine triphosphate (ATP) production. Absorption of dietary thiamine occurs in the small intestine by a saturable, carrier-mediated active process at low doses (oral intake less than 5 mg) and by a passive diffusion process at high doses (Hrubša et al. 2022; Smithline et al. 2012), whereas thiamine and thiamine pyrophosphate produced by gut microbiota are actively absorbed in the colon (Hrubša et al. 2022; Wan et al. 2022). A high daily oral thiamine dose gives a very high thiamine plasma level (Smithline et al. 2012), which may induce passive diffusion of thiamine into the cells with a secondary boost of mitochondrial ATP production. Malnutrition in patients with IBD is well known, however thiamine deficiency is not common and therefore routinely assessment of thiamine and other micronutrient deficiencies (vitamin K, selenium, vitamin A, vitamin C, zinc, vitamin B6) are not recommended (Massironi et al. 2023; Bischoff et al. 2023; Maaser et al. 2019).

In the TARIF clinical trial (ClinicalTrials study identifier NCT036347359), we found that high doses of oral thiamine hydrochloride administered for 4 weeks were able to reduce fatigue levels in patients with quiescent IBD and chronic fatigue (Bager et al. 2021). The TARIF study was a randomised, double-blinded, placebo-controlled crossover trial including patients with quiescent IBD and chronic fatigue and no other explanation for fatigue. Weight- and gender-adjusted doses given in the study ranged between 600 and 1800 mg per day. Side effects to high-dose thiamine were few and temporary.

In the TARIF study, blood samples were collected for analyses of B-vitamins and metabolites. In addition to samples from 40 patients with quiescent IBD and chronic fatigue, blood samples from 20 matched patients with quiescent IBD and no fatigue were collected. This allowed us to:

1. compare the plasma levels of selected B vitamins, related vitamers and metabolites between chronic fatigued patients with quiescent IBD and non-fatigued patients with quiescent IBD;
2. examine plasma levels of selected B-vitamins, related vitamers and metabolites between chronic fatigued patients with quiescent IBD with or without positive effect of high doses of oral thiamine.

## Material and methods

### Study design

The TARIF study included 40 adult patients with quiescent IBD and chronic fatigue consecutively from the outpatient clinic at Aarhus University Hospital, Denmark. Eligible patients had had a diagnosis of IBD for more than 12 months and had disease in remission. Fatigue severity were assessed using the Inflammatory Bowel Disease-Fatigue Questionnaire (IBD-F) section I. Patients with a fatigue score > 12 and fatigue duration > 6 months were included (Czuber-Dochan et al. 2014; Bager et al. 2018). Patients with anemia, iron deficiency, folate acid deficiency, vitamin-B12 deficiency, or vitamin-D deficiency were excluded. We excluded pregnant women and patients with co-morbidity that could explain a high level of fatigue (e.g. cancer, chronic kidney disease, chronic heart disease, diabetes). Patients were allocated 1:1 to either; (1) high-dose oral thiamine for 4 weeks, followed by 4 weeks of washout, followed by 4 weeks of oral placebo; or (2) oral placebo for 4 weeks, followed by 4 weeks of washout, followed by 4 weeks of high-dose oral thiamine.

**Fig. 1:**
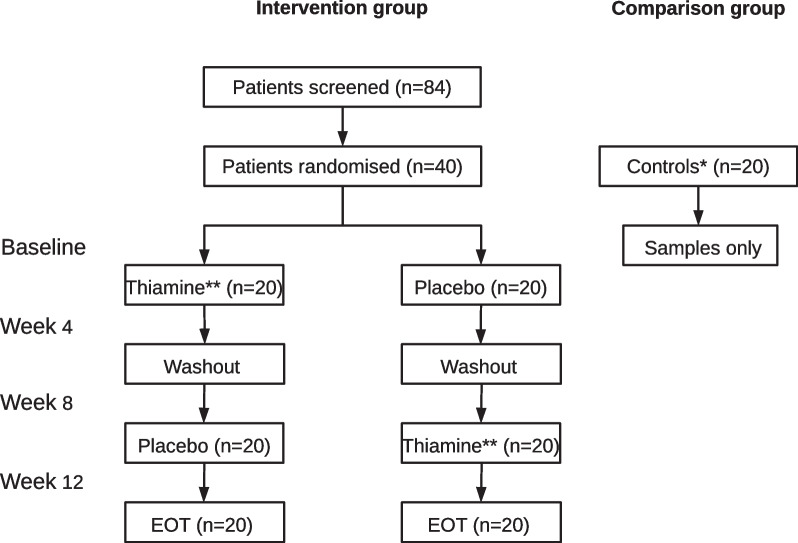
TARIF trial profile. EOT, End of trial; * Controls had quiescent IBD and a fatigue score ≤ 12 on the IBD-F scale; ** High dose (600–1800 mg day) for 4 weeks

A comparison group was included parallel to inclusion in the intervention study. We included 20 controls with quiescent IBD and no fatigue (fatigue score ≤ 12 on the IBD-F scale), matched on gender, age, and IBD disease type (Fig. 1, Table 1). Blood samples were drawn, and the patients answered questionnaires regarding fatigue at each study visit (baseline, week 4, week 8, and week 12). The controls had only drawn blood samples and answered fatigue questionnaires at baseline.

**Table 1:** Baseline characteristics for 40 chronic fatigued and 20 non-fatigued patients with quiescent inflammatory bowel disease (IBD)

	Patients with chronic fatigue (n = 40)	Patients with no fatigue (n = 20)	p-value
Age, years; *mean (SD)*	37.3 (12.7)	33.0 (10.2)	0.19
Sex, male; *n (%)*	5 (13)	3 (15)	0.79
Body weight, kg; *mean (SD)*	73.3 (17.7)	73.4 (14.6)	0.98
Crohns disease; *n (%)*	20 (50)	10 (50)	1.00
Ulcerative colitis; *n (%)*	20 (50)	10 (50)	1.00
Hemoglobin, g/dl; *mean (SD)*	8.4 (0.7)	8.4 (0.8)	0.94
CRP, mg/l; *median (IQR)*	4.0 (4.0–4.0)	4.0 (4.0–4.0)	0.98
Albumin, g/l; *mean (SD)*	40.0 (3.3)	39.2 (2.5)	0.59
Thiamine, nmol/l; *mean (SD)*	4.4 (4.0)	3.7 (2.6)	0.45
IBD-F I; *mean (SD)*	14.8 (1.9)	5.3 (2.1)	< 0.01

*IBD* inflammatory bowel disease, *SD* standard deviation, *CRP* C-reactive protein, *IQR* interquartile range, *IBD-F I* Inflammatory Bowel Disease-Fatigue Questionnaire section I

We assessed fatigue severity using the IBD-F questionnaire section I. It yields a score between 0 and 20 where higher score indicates more fatigue. Patients with a fatigue score > 12 were classified as being fatigued (Czuber-Dochan et al. 2014) as 12 was equivalent to the 95^th^ percentile for fatigue reported by a background population (Bager et al. 2018). A clinical important improvement of fatigue was defined as ≥ 3 points decrease in the IBD-F score.

The study was conducted according to the principles of the Helsinki declaration, and the protocol and informed consent forms were approved by the Ethics Committee in Central Denmark Region (j.no. 64207) and the Danish Medical Agency (EudraCT j.no. 2018-002324-17). Written informed consent was obtained from all participants.

### Blood samples

Blood samples were collected at the time points described above. All plasma samples were stored at − 80 °C and analyzed in one batch after study completion. Plasma were analyses for relevant B-vitamins and metabolites (listed in Table 1) by Bevital AS, Norway (http://bevital.no) (Midttun et al. 2009).

### Data analysis

Baseline data from fatigued patients and non-fatigued patients were compared. Changes in fatigue score before and after actual high dose thiamine treatment were calculated. Data from the group of patients with ≥ 3 points decrease in fatigue were compared to the group with < 3 points decrease.

Data were analyzed by nonparametric statistics, using the Mann–Whitney ranked sum test. Data are presented as the median and interquartile range (IQR). P-values < 0.05 were considered statistically significant. Data analysis was conducted in Stata (version 18.0, StataCorp, College Station, Texas).

## Results and discussion

Blood samples were available from all 60 IBD patients, i.e. 40 patients with chronic fatigue and 20 patients matched for gender and age and without fatigue. The median age was 35 years (IQR 25–45). Eight patients were men and 52 were women. Half of the patients had Crohn’s disease and half had ulcerative colitis (Table 1). In the TARIF study, 26 (65%) fatigued patients had ≥ 3 points decrease of fatigue score following thiamine treatment (Bager et al. 2021).

When comparing the baseline samples from the 40 patients with chronic fatigue with samples from the 20 patients without fatigue, only the concentration of flavin mononucleotide (FMN) a B2 vitamer, was different between the groups (Table 2). Fatigued patients had a statistically significantly lower level of FMN compared with non-fatigued patients. At baseline, the median (IQR) thiamine plasma concentrations were 2.9 (1.8–5.3) and 2.7 (2.0–5.3) nmol/L in patients with and without fatigue, respectively. For the patients with fatigue, baseline thiamine levels were not associated with a decrease in fatigue after the intervention.

**Table 2:** Plasma levels of B-vitamin, related vitamers, and metabolites at baseline

	Analysis	Patients with chronic fatigue (n = 40)	Patients with no fatigue (n = 20)	*p*-value
Vitamin B1	Thiamine (nmol/L)	2.9 (1.8 to 5.3)	2.7 (2.0 to 5.3)	0.97
	Thiamine monophosphate (nmol/L)	8.3 (5.4 to 9.8)	8.3 (6.0 to 11.0)	0.70
Vitamin B2	Riboflavin (nmol/L)	12.1 (6.9 to 18.5)	11.2 (7.7 to 19.7)	0.66
	Flavin mononucleotide (nmol/L)	11.1 (8.4 to 13.7)	14.2 (10.9 to 17.0)	0.02
Vitamin B3	Nicotinic acid (nmol/L)	368 (279 to 476)	372 (311 to 471)	0.72
	*N*1-methylnicotinamide (nmol/L)	361 (264 to 547)	439 (302 to 516)	0.66
Vitamin B6	Pyridoxal 5ʹ-phosphate (nmol/L)	52.0 (36.0 to 86.4)	41.6 (33.8 to 56.2)	0.16
	Pyridoxal (nmol/L)	12.6 (9.6 to 21.0)	9.5 (8.4 to 12.8)	0.06
	4-Pyridoxic acid (nmol/L)	19.7 (12.0 to 38.6)	12.6 (11.6 to 26.5)	0.32
Tryptophan metabolites	Kynurenine (μmol/L)	62.8 (55.1 to 73.3)	60.9 (52.7 to 65.9)	0.26
	Kynurenic acid (nmol/L)	1.6 (1.3 to 1.9)	1.6 (1.3 to 1.7)	0.86
	Anthranilic acid (nmol/L)	44.8 (31.9 to 57.8)	50.4 (44.1 to 67.0)	0.07
	3-Hydroxykynurenine (nmol/L)	43.0 (36.3 to 57.1)	43.0 (34.1 to 57.3)	0.99
	Xanthurenic acid (nmol/L)	17.1 (12.8 to 23.6)	20.0 (12.5 to 22.1)	0.55
	3-Hydroxyanthranilic acid (nmol/L)	9.9 (7.8 to 12.7)	10.1 (9.0 to 11.3)	0.83
	Picolinic acid (nmol/L)	40.1 (29.9 to 50.4)	42.8 (29.7 to 57.4)	0.47
	Quinolinic acid (nmol/L)	38.9 (23.6 to 47.3)	43.3 (33.6 to 58.3)	0.21
Nicotine metabolites	Cotinine (nmol/L)	124 (79 to 152)	111 (91 to 194)	0.95
	Trans-3ʹ-hydroxycotinine (nmol/L)	0.31 (0.20 to 0.46)	0.25 (0.16 to 0.39)	0.34
Other	Cystathionine (μmol/L)	14.5 (10.8 to 18.8)	13.5 (10.3 to 17.1)	0.58
	Trigonelline (μmol/L)	0.69 (0.38 to 2.50)	1.02 (0.47 to 3.38)	0.44

Comparison of 40 chronic fatigued and 20 non-fatigued patients with quiescent inflammatory bowel disease (IBD). Results are showed as median and interquartile range in parentheses

In the 40 patients with chronic fatigue, changes in plasma levels of B-vitamins and metabolites directly after high dose thiamine treatment for 4 weeks were assessed. Treatment with thiamine caused a 100-fold increase in plasma thiamine, but only minor changes in the other biomarkers. Subsequently, we compared the changes between patients who reported a positive effect on fatigue on ≥ 3 points on the IBD-F scale (n = 26) with patients who did not experience changes in fatigue scores (n = 14). Patients who reported effect on fatigue following high dose thiamine had a statistically significantly drop in the median level of riboflavin directly after treatment (p = 0.01), but as shown in Table 3, other biomarkers showed no statistically significant changes between responders and non-responders.

**Table 3:** Changes in plasma levels of B-vitamin, related vitamers, and metabolites directly after high dose thiamine treatment for 4 weeks in 40 patients with quiescent inflammatory bowel disease (IBD) and chronic fatigue

	Analysis	Patients with effect after thiamine (n = 26)	Patients without effect after thiamine (n = 14)	*p*-value
Vitamin B1	Thiamine (nmol/L)	295.5 (141.2 to 506.1)	180.3 (53.9 to 408.9)	0.51
	Thiamine monophosphate (nmol/L)	13.0 (10.6 to 22.2)	18.4 (6.9 to 23.6)	0.85
Vitamin B2	Riboflavin (nmol/L)	− 1.6 (− 5.3 to 0.9)	1.9 (0.2 to 6.2)	0.01
	Flavin mononucleotide (nmol/L)	− 0.6 (− 2.7 to 1.4)	0.5 (− 0.7 to 1.2)	0.85
Vitamin B3	Nicotinic acid (nmol/L)	33 (− 12 to 77)	25 (− 37 to 87)	0.89
	*N*1-methylnicotinamide (nmol/L)	− 92 (− 267 to 137)	424 (− 72 to 141)	0.42
Vitamin B6	Pyridoxal 5ʹ-phosphate (nmol/L)	− 9.5 (− 21.9 to 1.5)	− 4.2 (− 15.8 to 1.3)	0.67
	Pyridoxal (nmol/L)	− 2.3 (− 8.9 to − 0.4)	− 0.4 (− 3.0 to 3.2)	0.10
	4-Pyridoxic acid (nmol/L)	− 1.6 (− 7.6 to 6.4)	− 0.3 (− 0.8 to 4.2)	0.95
Tryptophan metabolites	Kynurenine (μmol/L)	− 0.3 (− 9.9 to 3.1)	− 3.5 (− 7.4 to 3.4)	0.94
	Kynurenic acid (nmol/L)	0.1 (− 0.1 to 0.2)	0.3 (− 0.1 to 0.2)	0.99
	Anthranilic acid (nmol/L)	3.4 (− 4.1 to 22.9)	4.5 (− 0.4 to 13.0)	0.86
	3-Hydroxykynurenine (nmol/L)	3.7 (− 2.1 to 8.5)	0.1 (− 3.4 to 6.3)	0.44
	Xanthurenic acid (nmol/L)	2.6 (− 3.6 to 13.9)	0.8 (− 1.8 to 4.7)	0.73
	3-Hydroxyanthranilic acid (nmol/L)	0.4 (− 1.7 to 2.5)	0.0 (− 1.1 to 2.3)	0.53
	Picolinic acid (nmol/L)	1.9 (− 7.9 to 13.4)	− 1.5 (− 6.6 to 12.8)	0.83
	Quinolinic acid (nmol/L)	4.9 (− 6.3 to 14.4)	1.8 (− 3.4 to 9.1)	0.92
Nicotine metabolites	Cotinine (nmol/L)	− 19 (− 60 to 19)	− 2 (− 13 to 53)	0.15
	Trans-3ʹ-hydroxycotinine (nmol/L)	0.03 (− 0.06 to 0.14)	0.03 (− 0.05 to 0.10)	0.99
Other	Cystathionine (μmol/L)	− 0.1 (− 1.0 to 4.2)	0.1 (− 1.5 to 0.7)	0.44
	Trigonelline (μmol/L)	0.04 (− 1.57 to 2.34)	− 0.02 (− 0.07 to 0.89)	0.57

Comparison between the 26 IBD patients who reported effect on fatigue (fatigue decrease of ≥ 3 points i the IBD-F scale) after oral high dose thiamine treatment and the 14 IBD patients without effect. Results are showed as median and interquartile range in parentheses

## Discussion

This study aimed to explore the dynamics in plasma levels of B-vitamins and their related vitamers and metabolites in relation to chronic fatigue in patients with quiescent IBD. We found that the plasma level of FMN was lower in patients with chronic fatigue than in patients without fatigue. Furthermore, we found that changes in the plasma levels of riboflavin were significantly different between chronic fatigued IBD patients who had positive effect of oral high doses of thiamine and fatigued patients who did not have effect of high doses of thiamine.

The bioactive forms of riboflavin, FMN and flavin adenine dinucleotide (FAD), are essential for the energy production through ATP (Udhayabanu et al. 2017). This indicates that our finding of a lower level of FMN in quiescent IBD patients with chronic fatigue may reflects a causal relation. We found that the level of riboflavin decreased in patients who had effect of high dose thiamine treatment. The decrease may be related to an increased mitochondrial production of ATP mediated by the high doses of thiamine and a subsequent higher consumption of riboflavin and FMN. Whether specific supplementation with riboflavin or FMN may have positive effects on chronic fatigue in patients with quiescent IBD is a question for further research. Also, it has not been investigated if supplementation with riboflavin together with high doses of thiamine could boost the positive effect on fatigue.

Borren et al. investigated both serum metabolomics and the faecal microbiome in relation to fatigue in patients with quiescent IBD (Borren et al. 2021). In serum, the metabolites methionine, tryptophan, proline, and sarcosine were found to be significantly depleted in patients with fatigue. The researchers found that fatigued IBD patients had a less diverse microbiome with reduced numbers of butyrate-producing bacterial species compared to non-fatigued patients. Others found that low levels of butyrate are associated with chronic fatigue. Intestinal butyrate production directly depends on the presence of both thiamine and riboflavin (Soto-Martin et al. 2020). Because thiamine is important for microbial growth (Wan et al. 2022) and possibly affected the number of riboflavin-producing microbial strains, the high oral intake of thiamine in our study may both have changed the microbiota directly and served to facilitate butyrate production.

The mechanisms of B-vitamins in IBD in general and in relation to fatigue is still not fully explored. As thiamine is important for the growth of gut bacteria and studies have shown associations between fatigue and microbiota in healthy controls compared to patients with IBD; further fatigue-studies including B-vitamins and microbiota could add more knowledge to this topic (Borren et al. 2021; Kim et al. 2019). As fatigue is prevalent across many different autoimmune diseases comparisons of patients with fatigue across diseases could also be interesting to study. We believe that this study has added a small puzzle piece to the body of evidence regarding fatigue, IBD and B-vitamins. Based on our findings it could be interesting to investigate the role of riboflavin in the treatment of fatigue. This could be alone or in combination with thiamine, ideally in a blinded randomised crossover trial.

The TARIF study design was robust and showed significant effect on fatigue of high doses of oral thiamine. However, both the study itself and the post analysis of B-vitamins and related vitamers has some limitations. The study included mostly females. This is no surprise as fatigue is more prevalent in females than in males with IBD (Bager et al. 2012). The sample size appeared to appropriate as the primary results were clear. Furthermore, the control group were closely matched the intervention group. However, a larger sample may have revealed more significant results in this B-vitamin sub-study. This could be considered in future studies. The strength of our study was the longitudinal design including repeated measurements of both fatigue and blood samples. We could have investigated other factors that may influence fatigue and vitamins (diet, sleep pattern, physical activity etc.). However, we that found B-vitamins and vitamers would be the most relevant factors in an intervention study including high dose thiamine exposure.

In conclusion, riboflavin and the metabolite FMN were found to be associated with chronic fatigue in patients with quiescent IBD. Levels of other B vitamins, related vitamers, and metabolites were not found to be significantly different between the investigated groups or related to the thiamine intervention.

## Data Availability

The datasets used and/or analysed during the current study are available from the corresponding author on reasonable request.

## Abbreviations

ATP: Adenosine triphosphate
CRP: C-reactive protein
EOT: End of trial
FAD: Flavin adenine dinucleotide
FMN: Flavin mononucleotide
IBD: Inflammatory bowel disease
IBD-F: Inflammatory Bowel Disease-Fatigue Questionnaire
IQR: Interquartile range
SD: Standard deviation
